# From Neighboring Behavior to Mental Health in the Community: The Role of Gender and Work-Family Conflict

**DOI:** 10.3390/ijerph16122101

**Published:** 2019-06-13

**Authors:** Zhenduo Zhang, Li Zhang, Xiaoqian Zu, Tiansen Liu, Junwei Zheng

**Affiliations:** 1 School of Management, Harbin Institute of Technology, Harbin 150001, China; 17B910059@stu.hit.edu.cn (Z.Z.); 15B910002@hit.edu.cn (X.Z.); 2 School of Economics and Management, Harbin Engineering University, Harbin 150001, China; tiansen0328@hotmail.com; 3 Department of Construction Management, Kunming University of Science and Technology, Kunming 650500, China

**Keywords:** neighboring behavior, mental health, gender, work-family conflict, community

## Abstract

This research emphasizes the potential influences of social community environments on low-income employees’ mental health. Using a two-wave panel design, we collect 218 matched data from low-income employees in Harbin City, China. We developed a moderated mediation model to test our hypotheses with the following significant results: (1) neighboring behavior, defined as both giving and receiving various kinds of assistance to and from one’s neighbors, positively influenced mental health; (2) work-family conflict mediated the relationship between neighboring behavior and mental health; (3) gender moderated the influences of neighboring behavior on mental health, such that neighboring behavior had a stronger positive influence on mental health for females than for males; (4) gender moderated the mediating effect of work-family conflict; that is, the positive influences of neighboring behavior were stronger for female employees than for male employees. This research explores the mechanism and boundary conditions of the relationship between neighboring behavior and mental health. In practice, community managers support community social workers by organizing community-building social activities and supportive programs to enhance residents’ neighboring behavior.

## 1. Introduction

Mental health is currently a hot topic of discussion, both practically and theoretically, especially for low-income employees [1,2]. Due to the financial fragility and stress associated with living at or near poverty, low-income employees may suffer a higher than average risk of developing mental health problems while having access to fewer resources [3]. Previous research has examined the antecedents of their mental illness from individual, family, and workplace characteristics [4,5,6]. However, there has been little exploration into the potential influences of neighboring behavior on individuals’ mental health.

In addition to a physical community environment, Perkins et al. [7] put forward the concept of a social community environment to examine the extent to which individuals participate in their physical communities. Neighboring behavior refers to both giving and receiving various kinds of assistance to and from neighbors [7], such as offering a neighbor advice on personal problems (given neighboring behavior) and being helped by your neighbors in an emergency (received neighboring behavior). Neighboring behavior is a core dimension of social community environments [7], frequently complementing a person’s network existing outside his or her residential neighborhood environment [8]. The close spatial location of neighbors makes them uniquely poised to aid each other, both practically and emotionally [8]. It enhances residents’ social cohesion and helps them to acquire qualified social capital, which has been examined as a powerful amplifier of personal social functioning [9].

Work-family conflict refers to a form of inter-role conflict in which stress from work and stress from family play mutually incompatible roles in some respect [10]. It derives from both job and family stress, leading to mental illness, such as depression, exhaustion, and anxiety [11], which is further associated with suicidal behavior [12]. As a beneficial context, neighboring behavior facilitates social relationships of low-income employees, providing them with valuable resources to cope with work-family conflict, and to further improve their mental health. In this vein, the study here introduces work-family conflict as a mediator to answer our first research question, “how does neighboring behavior enhance low-income employees’ mental health?”

Previous research has revealed that gender plays a vital role in shaping individuals’ feelings of work-family conflict [13]. For females, especially in China, the family role is typically more salient [14]. Moreover, given the increasing competition in the workplace, it might be expected that females would have higher risks of experiencing work-family conflict than males [14,15]. Therefore, neighboring behavior can be inferred to be more important for females than for males [3]. Expanding upon these findings, we introduce gender as a moderator in the indirect relationship between neighboring behavior and mental health through work-family conflict to answer the second question of this study, “how does neighboring behavior influence low-income employees’ mental health?”

**Figure 1:**
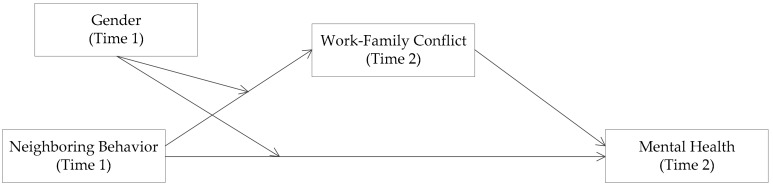
Conceptual Model.

To address these questions, we applied a two-wave design to test our conceptual model (Figure 1) based on the conservation of resources (COR) theory. In so doing, our research has three potential contributions to current mental health literature. First, this research expands the scope of previous research concerning the influences of social support by adopting neighboring behavior (including both given and received neighboring behavior) as an antecedent to low-income employees’ mental health, deepening our understanding of social community environmental impacts on personal psychological outcomes. Second, this research introduces work-family conflict as a mediator and unveils the underlying mechanism of the relationship between neighboring behavior and mental health. Finally, our research offers a comprehensive understanding of the influences of neighboring behavior by exploring the moderating role of gender. Our research highlights the importance of taking individual factors into consideration when exploring how social community environments affect psychological outcomes.

## 2. Literature Review and Hypothesis Development

### 2.1. Mediating Role of Work-Family Conflict

#### 2.1.1. Neighboring Behavior and Work-Family Conflict

Unger and Wandersman [8] address the importance of differentiating the influences of social community environment from physical community environment on individuals’ psychological outcomes. Moreover, they used bidirectional—both received and given neighboring behavior—rather than only received behavior, to represent social community environment [7], providing evidence for the positive influences of neighboring behavior on social cohesion and decreased community crime rates [7].

From the perspective of received neighboring behavior, low-income employees received social support from their neighbors, which provided them with the necessary resources to cope with work-family conflict [16]. Low-income employees may garner social support from social networks outside the work and family domains [3]. Ecological systems theory suggests that community microsystems are part of social networks, which can offer resources to work and family systems [17]. Low-income employees experience stress both from life and work domains due to their fragile financial situations and fierce work competition [3]. Therefore, neighboring support is necessary for low-income employees to take care of responsibilities and to respond to stressful experiences in both work and life domains [18]. Research has indicated that neighboring support can decrease work-family conflict for low-income employees [3,19]. From the perspective of given neighboring behavior, low-income employees may experience enhanced self-evaluations, which would in turn help them to cope with work-family conflict [20,21,22]. Helping neighbors can nurture low-income employees’ inherent sense of well-being, thereby increasing their self-evaluations [23]. High self-evaluations drive low-income employees to appraise events in work and family domains as more positive [24]. Those with high self-evaluations tend to seek situations advantageous to enhancing positive fulfillment in work and family roles, and tend to avoid negative situations leading to conflict between work and family roles [24]. Research has revealed that self-evaluation is negatively correlated with work-family conflict [25] and positively correlated with psychological well-being [26].

Moreover, drawing on the conservation of resources (COR) theory, individuals are motivated to protect and gain personal resources [27]. Given neighboring behavior is an investment of possessed resources (like time, knowledge or social support) to earn future resources [28], and received neighboring behavior is an approach of resources acquisition. Gained resources enable low-income employees to resolve the problems triggered by stressful circumstances and help them recover from the negative emotions associated with resource loss [27]. Integrating the arguments and COR theory above, neighboring behavior promotes the social cohesion of low-income employees in the community [29], providing them with important social resources through both giving and receiving of help between neighbors to create positive results in both family and work domains [3], therefore decreasing their work-family conflict. Thus, we hypothesized the following:

**Hypothesis** **1.** *Neighboring behavior is negatively correlated with work-family conflict.*

#### 2.1.2. Work-Family Conflict and Mental Health

Work-family conflict occurs when efforts to fulfill work role demands interfere with one’s ability to fulfill family demands and vice versa [10]. Substantial research has revealed the detrimental influences of work-family conflict on mental health [30,31].

Previous research has adopted social identity theory to explain the relationship between work-family conflict and mental health [32]. Individuals tend to spend considerable time and energy on constructing and maintaining desired identities [33]. If self-identifying activities are impeded, people tend to experience damaged self-images [32]. Work-family conflict can be regarded as an interruption that has potential disadvantageous implications for low-income employees on meeting family and work-related responsibilities [34], thereby undermining their family—and work—related self-images. Under such context, low-income employees are more likely to experiences high level psychological stress because work and family self-images or roles are two basic components of adult identity [35]. Thus, work-family conflict negatively affects mental health.

The conservation of resources theory posits that individuals are motivated to protect and maintain valuable personal resources [27]. Psychological stress arises from continuous loss of resources [27]. When experiencing a spiraling loss of resources, individuals struggle to protect resources [36]. However, low-income employees living in or near the poverty line have fewer resources to cope with work-family conflict [3]. Under such contexts, low-income employees are more likely to be trapped in resource exhaustion, which has been examined as a predictor of mental illness.

Thus, we arrive at our second hypothesis:

**Hypothesis** **2.** *Work-family conflict is negatively related to mental health.*

According to COR theory, people who possess resources are more likely to cope with and withstand the loss of resources [27,37]. When individuals experience resources loss (e.g., work-family conflict), they might experience stress or depression [38]. Mental health can be optimized under the condition of low work-family conflict [39]. Combined with the theoretical basis and above hypotheses, neighboring behavior offers low-income employees necessary social support and increased self-evaluations, which help them cope with work-family conflict. Subsequently, decreased work-family conflict will result in a reduced negative impact on low-income employees’ work- and family-related self-images, as well as reduced depletion of valuable resources, an overall reduced negative impact on mental health. Hence, we arrive at our third hypothesis:

**Hypothesis** **3.** *Neighboring behavior has a significant indirect effect on mental health through work-family conflict.*

### 2.2. Moderating Role of Gender

The existence of gender differences is strongly evidenced in mental health literature [40]. Female employees are more likely to experience psychological stress because they tend to experience more stress from demands than do their male counterparts from equivalent demands [41]. Neighboring behavior is a basic way to maintain social relationships and enhance social cohesion [7,29], which we expect to be more important for the mental health of females than for the mental health of males.. COR theory posits that the values of resources vary depending on individual preferences [27]. For female low-income employees, they assign higher priority to their family roles and are more likely to experience conflicts between work and family roles [42]. With the increasing social resources derived from neighboring behavior, females are more likely to adapt to the gender role norms [42], therefore decreasing their experiences of work-family conflict. Thus, we arrive at our fourth hypothesis:

**Hypothesis** **4.** *Gender moderates the relationship between neighboring behavior and mental health, such that the positive relationship is stronger for females than for males.*

Social support has long been examined as a barrier to the negative influences of work-family conflict [43]. Drummond et al. (2017) found a moderating role of gender to the influences of both family and work support on psychological outcomes [42]. The individual differences and sources of resources may shape individuals’ coping strategies with work-family conflict [44]. For females, social support has a stronger impact on decreasing work-family conflict. Neighboring behavior can be regarded as a way to acquire composite community resources [7]. Both receiving help from neighbors and giving help to neighbors offers low-income employees the necessary resources (social support and self-evaluations) to cope with work-family conflict. Thus, on the basis of previous research concerning the relationship between social support and work-family conflict, we adopted gender as a moderator.

Eby et al. (2005) posited that both gender difference and gender role issues were essential to fully understand the work-family interface [45]. From the traditional Chinese gender role perspective, low-income female employees were expected to undertake responsibilities in both work and family domains [14], whereas the responsibilities of low-income male employees were limited primarily to the workplace. Particularly in desirable careers, female employees invest more time and energy than their male peers performing the same jobs [46]. Besides, the family role is more salient for female employees, who prioritize domestic duties over workplace demands [14]. Thus, female employees are likely to experience greater work-family conflict and are thus more responsive to the valuable resource of neighboring behavior, which may be more important for female employees than for male employees. Thus, we arrive at our fifth hypothesis:

**Hypothesis** **5.** *The relationship between neighboring behavior and work-family conflict is moderated by gender, such that the negative relationship is stronger for females than for males.*

Given that the family role is more salient for low-income female employees than for low-income male employees, neighboring behavior plays a more important role in decreasing work-family conflict, and thereby improving mental health, for females than for males. In Hypothesis 4 and 5, we theorized that neighboring behavior and gender would interactively predict work-family conflict and mental health, respectively. In Hypothesis 4, we argued that work-family conflict would play a mediating role between neighboring behavior and mental health. Thus, we expect that work-family conflict will mediate the interactive effect of neighboring behavior and gender on mental health (i.e., a conditional indirect effect [47]), then we arrive at our sixth hypothesis:

**Hypothesis** **6.** *Gender moderates the mediating role of work-family conflict on the relationship between neighboring behavior and mental health, such that neighboring behavior has a stronger impact on mental health through work-family conflict reduction for female employees than for male employees.*

## 3. Method

### 3.1. Procedures and Samples

Using a 2018 public occupancy document concerning government-subsidized housing from the Urban and Rural Planning Department of Harbin City, Heilongjiang Province, China, we developed a random sample pool of 317 low-income employees whose incomes fall below 2287 Chinese Yuan per month, which is the average monthly income for the city’s residents in the previous year in Heilongjiang Province. All randomly selected participants were at least 18 years of age, working regular, full-time jobs of at least 40 h per week, and lived in their current communities for at least 1 year. All individual participants in this study have signed the informed consent. For participants who have finished questionnaires both at time 1 and time 2, they were rewarded by 20 RMB (≈2.59 Euros).

**Figure 2:**
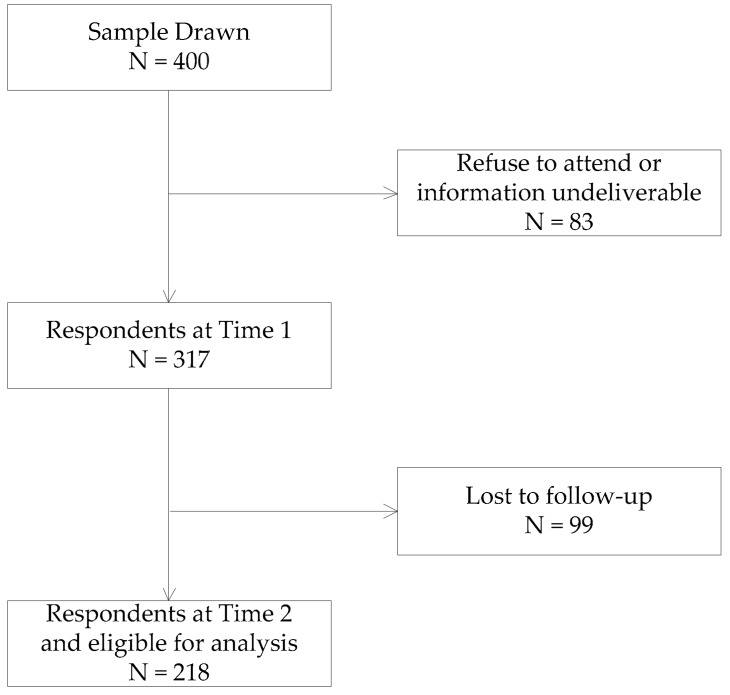
Flow of the study samples included in the current study.

We adopted a two-wave panel design and distributed our questionnaire on 1st November 2018 and 16th December 2018, respectively, with a five-week time span to better infer the casual relationship between our focal variables. In the first wave, we collected demographic information and neighboring behavior. In the second wave, which started five weeks after the end of the first wave, we collected work-family conflict and mental health. Social workers serving the chosen communities distributed our questionnaire after receiving training about the questionnaire. They sent hard copies of the questionnaires directly to participants and assisted with filling out the questionnaires. Finally, a total of 218 matched questionnaires were collected, with an effective response rate of 69%. The participants were employed in a variety of industries, including construction, manufacturing, and electronics, ensuring the repetitiveness of our samples. Males accounted for 41.3% of the participants, and 78.4% of the participants were married. The proportion of participants holding a bachelor’s degree or above was 18.8%. The average age was 41.43 ± 9.47 years, and the average years of work experience was 21.96 ± 9.47. Figure 2 presents a flow chart of the study population.

### 3.2. Measures

All of the items in our study were originally developed in English and translated into Chinese following a common back-translation procedure [48].

Neighboring behavior was measured using 10 items developed by Perkins et al. [7]. The scale included two dimensions: received and given neighboring behavior. Sample items of this scale were “receive aid from your neighbor in an emergency” (Received neighboring behavior) and “offer a neighbor advice on a personal problem” (Given neighboring behavior). It was a five-point Likert scale (1 = low frequency; 5 = high frequency). To test its validity of Chinese version, we conducted confirmatory analysis and the result showed the acceptable model fit (χ^2^(32) = 75.59, RMSEA = 0.08, RMR = 0.05, CFI = 0.96). The Cronbach’s α of this scale was 0.90.

Mental health was assessed by a 12-item general health questionnaire (GHQ) (Goldberg and Hiller [49]) which was validated by Gao [50] in Chinese sample populations. It contains three sub-dimensions: “anxiety”, “social dysfunction”, and “loss of confidence”. Sample items were “sleep loss due to worrying” (Anxiety), “ability to concentrate” (Social dysfunction) and “thoughts of self-worthlessness” (Loss of confidence). This scale was adapted into a five-point Liker scale (1 = strongly disagree; 5 = strongly agree). We used a reversed scoring of this scale in the current study; the higher the score, the better the respondent’s mental health. This scale yielded a Cronbach’s α of 0.96.

Work-family conflict was measured by 8 items developed by Grzywacz and Marks [51]. The scale can be divided into “family interference with work” and “work inference with family”. The scale was measured by a 5-point Likert scale (1 = never; 5 = all the time). Two sample items were “stress at work makes you irritable at home” and “stress at home makes you irritable at work”. This scale was previously used in Chinese samples and showed acceptable reliability [52]. The Cronbach’s α for this scale was 0.89.

Control variables: On the basis of previous research, we adopted gender (0 for male and 1 for female), age (in years), education (1 = junior school or below; 2 = senior school; 3 = college; 4 = bachelor’s degree; 5 = master’s degree or above), and marital status (0 for married and 1for single) [42,53]. Considering the high correlation of work experience with age (r = 0.96, *p* < 0.01), we did not include work experience in our regression model in order to rule out the potential multicollinearity.

## 4. Results

### 4.1. Confirmatory Factor Analysis

Given the self-reported questionnaire we used in the current study, we first conducted confirmatory factor analysis to examine the potential common method variance (CMV), using Mplus@7.4 (Muthén & Muthén, CA, USA). Because our focal variables (neighboring behavior, work-family conflict, and mental health) all had subdimensions, we adopted CFA with second order latent variables. For example, items were first loaded on “family interference with work” and “work interference with family”, followed by the two subdimensions on the latent variable “work-family conflict”. Results in Table 1 showed that our three-factor conceptual model had a better fit (χ^2^(395) = 922.60, RMSEA = 0.07, RMR = 0.046, CFI = 0.92) than any other alternative model, which supported the CMV in our research.

### 4.2. Descriptive Statistics 

Table 2 provides the descriptive statistics and correlations of all study variables. Neighboring behavior negatively correlated with work-family conflict (r =−0.47, *p* < 0.01) and positively correlated with mental health (r = −0.49, *p* < 0.01). Work-family conflict negatively correlated with mental health.

Further, we conducted an independent T-test and one-way ANOVA test based on demographic variables, the results of which are shown in Table 3. Focal variables showed no significant differences between female and male. Married low-income employees had higher scores of neighboring behaviors than single employees (t = −2.46, *p* < 0.05). Higher education levels correlated with higher neighboring behavior (F = 6.24, *p* < 0.01), better mental health (F = 12.94, *p* < 0.01), and lower work-family conflict (F = 22.27, *p* < 0.01).

### 4.3. Regression Results

We used SPSS@21(IBM Corp., Armonk, NY, USA) and Process Macro to test the hypotheses. Table 4 shows the results of model 1 indicating that neighboring behavior negatively correlated with work-family conflict (b = −0.43, *p* < 0.01). In model 3, neighboring behavior positively correlated with mental health (b = 0.45, *p* < 0.01). Model 4 examined neighboring behavior and work-family conflict in the same regression model, showing a significant relationship between neighboring behavior and mental health (b = 0.35, *p* < 0.01), and a significant influence of work-family conflict on mental health (b = −0.25, *p* < 0.05). Also, the bootstrapping test (Table 5) showed work-family conflict played a significant mediating role (Effect = 0.14, 95%CI = [0.07, 0.23]), supporting Hypotheses 1, Hypothesis 2, and Hypothesis 3.

In model 2, the interactive item of neighboring behavior with gender correlated with lower work-family conflict (b = −0.38, *p* < 0.01). In model 5, the interactive item of neighboring behavior with gender correlated with better mental health (b = 0.44, *p* < 0.05).

**Figure 3:**
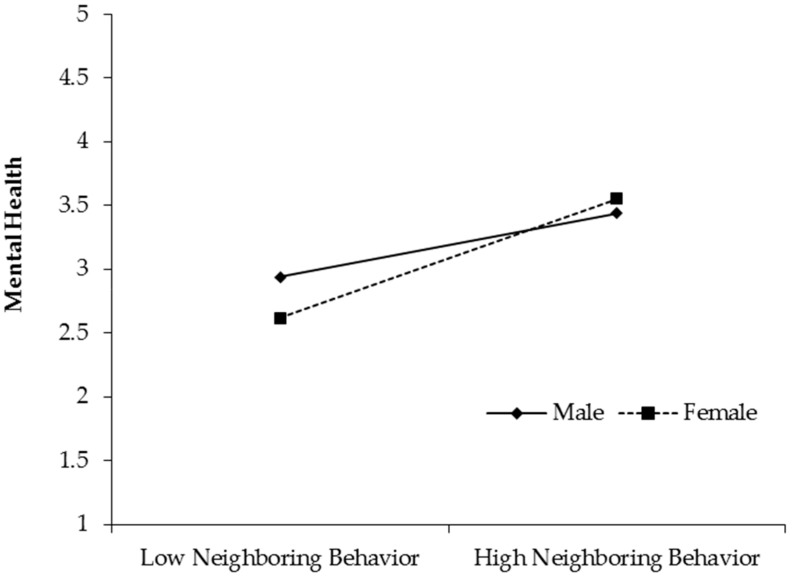
Moderating role of gender in the relationship between neighboring behavior and mental health.

To directly examine the moderating role of gender, we followed the suggestions of Aiken and West (1991) [54]. In Figure 3, neighboring behavior had a stronger influence on mental health for female employees (Effect = 0.79, 95% CI = [0.58, 1.00]) than for males (Effect = 0.42, 95% CI = [0.20, 0.65]). The difference was 0.37, and 95% confidence interval was [0.06, 0.67]. Hypothesis 4 was supported.

**Figure 4:**
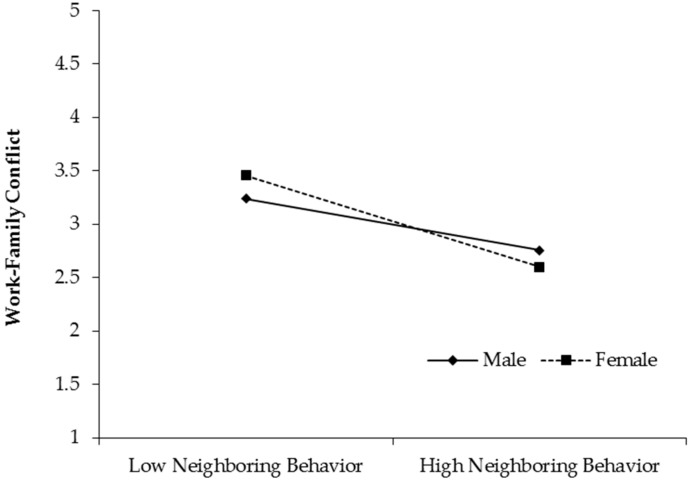
Moderating role of gender in the relationship between neighboring behavior and work-family conflict.

Likewise, in Figure 4, the indirect effect of neighboring behavior on mental health through work-family conflict was stronger for female employees (Effect = 0.18, 95%CI = [0.09, 0.29]) than males (Effect = 0.10, 95%CI = [0.04, 0.19]). The difference was 0.08, and 95% confidence interval was [0.01, 0.19]. We also conducted a simple slope test for the moderating effect of gender on the relationship between neighboring behavior and work-family conflict. For females, neighboring behavior had a stronger impact on work-family conflict (Effect = −0.72, 95%CI = [−0.93, −0.52]) than for males (Effect = −0.40, 95%CI = [−0.62, −0.18]). The difference was 0.32, and 95% confidence interval was [0.03, 0.61]. Hypothesis 5 was supported.

## 5. Discussions

### 5.1. Theoretical Implications

This study collected data from 218 low-income employees through a two-wave panel design. Through moderated mediation analysis, this study found that neighboring behavior could enhance low-income employees’ mental health through decreasing their work-family conflict. Gender moderates this mediated relationship. For females, neighboring behavior has a more positive influence on mental health via a greater reduction in work-family conflict. This research has three contributions to mental health literature. 

First, by incorporating neighboring behavior, this research extends the antecedents of low-income employees. Previous research has examined the positive influences of community support on mental health [55]. However, the development and maintenance of a social relationship is a bidirectional process [56]. Examining only one component of support yields an incomplete view of the influence of neighboring behavior on psychological outcomes [7,56]. Receiving neighboring behavior may provide low income employees with external resources to cope with stressful affairs [3]. Helping neighbors may enhance low income employees’ self- evaluations, a contributing factor to decreased stress and improved mental health [22]. This study examined the impacts of neighboring behavior, a composite concept involving bidirectional neighboring activities, on mental health, enlarging the scope of mental health literature. Further, our research advances COR theory by enlarging the scope of resources through examining the positive influences of both given and receiving neighboring behavior on enhancing mental health.

Second, by examining the mediating role of work-family conflict, this research uncovers the underlying mechanism through which neighboring behavior impacts mental health. Low-income employees experience stress both from work and family domains [3]. Due to fragile financial situations and competition for work, low income employees are more likely to be trapped in a vicious cycle between work-family conflict and psychological stress, leading to mental health decline [57]. The mitigation of work-family conflict requires sufficient external and internal resources [58]. Neighboring behavior could facilitate social cohesion, leading low-income employees to share in the collective energy and support system of his community when his own are exhausted [29]. This study found that neighboring behavior helped low income employees in breaking the vicious cycle of work-family conflict with psychological stress, thereby enhancing their mental health, which introduced an important approach to fostering mental health.

Third, by exploring the moderating role of gender, this study clarifies the boundary condition under which neighboring behavior is more or less beneficial for mental health. Work-family conflict arises from the work and family role stress [59]. In traditional gender roles, females are expected to undertake most of the family responsibilities [60]. However, low-income female employees additionally devote themselves to work-related responsibilities. Females also tend to appraise stressful events as more disadvantageous and are more likely to turn to social relationships in order to acquire resources to cope with such stress [61]. Therefore, neighboring behavior plays a more important role for females in decreasing work-family conflict and enhancing mental health. Our research sheds light on gender differences in the effect of neighboring behavior and highlights the necessity to take personal characteristics into consideration in the mental health literature.

### 5.2. Practical Implications

Our research has some practical implications to enhance low income employees’ mental health. Community managers should support community social workers by organizing community-building social activities and supportive programs. Previous research has provided us with beneficial intervention programs in enhancing social cohesion and decreasing residents’ mental illness. For instance, Hardiman and Segal called for establishing self-help agency (SHA) to foster the enhancement of peer-oriented social networks and lead to the experience of shared community [62]. In this process, skilled social workers are acting as teachers delivering basic psychological and physical health knowledge to members and mentors to ensure members’ self-help skill mastery. Social workers can thus serve as a bridge between the low income-families and social resources to help low-income families integrate into their communities. Furthermore, social workers should provide differentiated services based on community members’ gender, age and marital status to help them reduce conflict and increase facilitation for well-being.

### 5.3. Limitations and Future Research

This study has some limitations and may provide suggestions for future research. For methodology, a two-wave panel design could provide this study with a methodological advantage to inferring the causal relationship between neighboring behavior and its outcomes (work-family conflict and mental health). However, we still cannot rule out reversed causal relationship [63]. Future research could provide interventions aimed at enhancing neighboring behavior, or could use a multi-wave cross lagged research design to develop a firm causal relationship between neighboring behavior and its outcomes. Further, our data were all collected through self-reported questionnaires, which may result in CMV. Although we provide evidence that the CMV is not significant in our research, future research could collect multi-source data to decrease the potential CMV, and to test the robustness of our results [64]. For example, future research may collect data from two interactive families and use an actor-partner interdependence model (APIM; [65,66]) to examine the influences of the interactive process between two families on low-income employees’ work-family conflict and mental health.

For content, neighboring behavior varies contingent upon age and gender [8,67,68]. However, our analysis showed no significant relationship between age and neighboring behavior (r = −0.01, n.s.), the same as the relationship between gender and neighboring behavior (r = 0.06, n.s). We further test the interactive effect of age with gender on neighboring behavior, but the parameter is still not significant (b = −0.01, n.s). The insignificant result may be partially resulted from the overall low-level neighboring behavior in our sample (mean = 1.87), which was also examined in the urban area in China by previous research [69]. Further, our research has mainly performed in urban low-income employees. Xu et al. has revealed there is a difference in neighboring behavior between urban and rural residents in China, showing a higher-neighboring behavior in the rural area than in the urban area [70]. Future research should incorporate rural low-income employees and examine how neighboring behavior shapes their work-family conflict and mental health, which may provide us with a complete view on the influences of neighboring behavior in China.

## Figures and Tables

**Table 1:** Confirmatory Factor Analysis.

Models	Variables	$\chi^{2}$	*df*	**△** $\chi^{2}$	RMSEA	RMR	CFI
Three-Factor	NB, WFC, GHQ	922.60	395		0.07	0.06	0.92
Alternative Model							
Two-Factor	NB+WFC, GHQ	935.18	397	12.42 **	0.08	0.08	0.91
Two-Factor	NB+GHQ, WFC	1318.08	397	395.48 **	0.10	0.15	0.85
Two-Factor	NB, WFC + GHQ	958.56	397	35.96 **	0.08	0.09	0.91
One-Factor	NB + WFC + GHQ	1296.81	398	374.21 **	0.10	0.11	0.85

Note. NB = Neighboring Behavior, WFC = Work-Family Conflict, GHQ = General Health Questionnaire. ** *p* < 0.01.

**Table 2:** Mean, Standard Deviance, Correlations.

	1	2	3	4	5	6	7	8
1.Gender	-							
2.Age	0.12	-						
3.Marital	0.08	−0.41 **	-					
4.Education	−0.08	−0.38 **	0.14 *	-				
5.Work Time	0.10	0.96 **	−0.42 **	−0.53 **	-			
6.Neighbouring Behavior (Time 1)	0.06	−0.01	0.17 *	0.24 **	−0.08	(0.90)		
7.Work-Family Conflict (Time 2)	0.05	0.28 **	−0.08	−0.44 **	0.37 **	−0.47 **	(0.89)	
8.Mental Health (Time 2)	−0.06	−0.08	0.07	0.38 **	−0.17 **	0.51 **	−0.49 **	(0.96)
Mean		41.43			21.96	1.87	3.01	3.13
SD		9.47			11.13	0.59	0.81	0.82

Note. * *p* < 0.05; ** *p* < 0.01. Values in the parenthesis are Cronbach’s α.

**Table 3:** Demographic Characteristic of Neighboring behavior, Work-Family Conflict and General Health.

Group	N (%)	Neighboring Behavior		Work-Family Conflict		Mental Health	
		Mean	T Value/F Value	Mean	T Value/F Value	Mean	T Value/F Value
Male	90 (41.3%)	1.83	−0.83	2.96	−0.80	3.19	0.91
Female	128 (58.7%)	1.90		3.05		3.09	
Married	171 (78.4%)	1.82	−2.46 *	3.04	1.15	3.10	−1.04
Single	47 (21.6%)	2.05		2.89		3.24	
Junior School or below	42 (19.3%)	1.86	6.24 **	3.22	22.27 **	2.94	12.94 **
Senior School	85 (39.0%)	1.73		3.30		2.86	
College	50 (22.9%)	1.78		3.05		3.21	
Bachelor	33 (15.1%)	2.20		2.06		3.81	
Master or above	8 (3.7%)	2.50		2.41		3.89	

Note. * *p* < 0.05; ** *p* < 0.01.

**Table 4:** Hierarchical Linear Regression Analysis Results.

Variables (N = 218)	Work-Family Conflict		Mental Health			
	Model 1	Model 2	Model 3	Model 4	Model 5	Model 6
Age	0.22 **	0.22 **	0.03	0.09	0.04	0.09
Marital	0.12	0.12 *	−0.03	0.00	−0.03	0.00
Education	−0.27 **	−0.26 **	0.29 **	0.22 **	0.28 **	0.22 **
Gender	0.02	0.02	−0.07	−0.06	−0.06	−0.06
Neighboring Behavior	−0.43 **	−0.06	0.45 **	0.35 **	0.04	0.03
Interaction		−0.38 *		−0.25 **	0.44 *	0.35
Work-Family Conflict						−0.23 **
F	24.94 **	21.92 **	22.03 **	21.617	19.74 **	19.32 **
R^2^	0.37	0.38	0.34	0.381	0.36	0.39
△R^2^		0.01*		0.039 **	0.02 *	0.03 **

Note. * *p* < 0.05; ** *p* < 0.01. Values in the table are standardized parameters.

**Table 5:** Results of Bootstrapping Test.

**Moderating Effect**	**Effect**	**SE**	**95%LLCI**	**95%ULCI**
Male	0.42	0.12	0.20	0.65
Female	0.79	0.11	0.58	1.00
Difference	0.37	0.16	0.06	0.67
**Moderated Mediation Model**	**Indirect Effect**	**SE**	**95%LLCI**	**95%ULCI**
Male	0.10	0.04	0.04	0.19
Female	0.18	0.05	0.09	0.29
Difference	0.08	0.05	0.01	0.19
**Path**	**Effect**	**SE**	**95%LLCI**	**95%ULCI**
Neighboring Behavior→Mental Health	0.48	0.09	0.30	0.65
Neighboring Behavior→Work Family Conflict→Mental Health	0.15	0.04	0.08	0.23

Note. SE, Standard Error. LLCI, Low level confidence interval; ULCI, Upper level confidence interval.
